# Genetic diversity of loquat (*Eriobotrya japonica*) revealed using RAD-Seq SNP markers

**DOI:** 10.1038/s41598-022-14358-9

**Published:** 2022-06-23

**Authors:** Yukio Nagano, Hiroaki Tashiro, Sayoko Nishi, Naofumi Hiehata, Atsushi J. Nagano, Shinji Fukuda

**Affiliations:** 1 Analytical Research Center for Experimental Sciences, Saga University, Saga, Japan; 2 The United Graduate School of Agricultural Sciences, Kagoshima University, Kagoshima, Japan; 3 Department of Biological Resource Science, Faculty of Agriculture, Saga University, Saga, Japan; 4 Agriculture and Forestry Technical Development Center, Nagasaki Prefectural Government, Nagasaki, Japan; 5 Faculty of Agriculture, Ryukoku University, Otsu, Japan; 6 Institute for Advanced Biosciences, Keio University, Tsuruoka, Yamagata Japan; 7 Center for Education and Research in Agricultural Innovation, Saga University, Saga, Japan

**Keywords:** Plant sciences, Plant breeding, Plant genetics, Plant molecular biology

## Abstract

Loquat (*Eriobotrya japonica*) have originated in southeastern China and spread as a cultivated plant worldwide. Many of the loquat genetic resources collected internationally are of unknown origin, and their genetic background requires clarification. This study analyzed the genetic diversity of 95 accessions by using Rad-Seq SNP markers. Data analysis broadly classified loquat into three groups: (1) Japanese and Chinese cultivars and some Japanese strains (wild plants that are not used for commercial cultivation), (2) Vietnamese, Israeli, Greek, USA, and Mexican cultivars and strains, and (3) other Japanese strains. Group 2 is cultivated mostly outside of East Asia and was clearly distinct from the other groups, indicating that varieties of unknown origin with genetic backgrounds different from those of Japanese and Chinese cultivars may have been introduced to Mediterranean countries and North America. Because Japanese and Chinese cultivars belong to group 1, the current Japanese cultivars are derived from genetic resources brought from China. Some of group 1 may have been introduced to Japan before excellent varieties were developed in China, while group 3 may have been indigenous to Japan that have not been introduced by human activities, or may have been brought to Japan by human activities from China.

## Introduction

**Figure 1:**
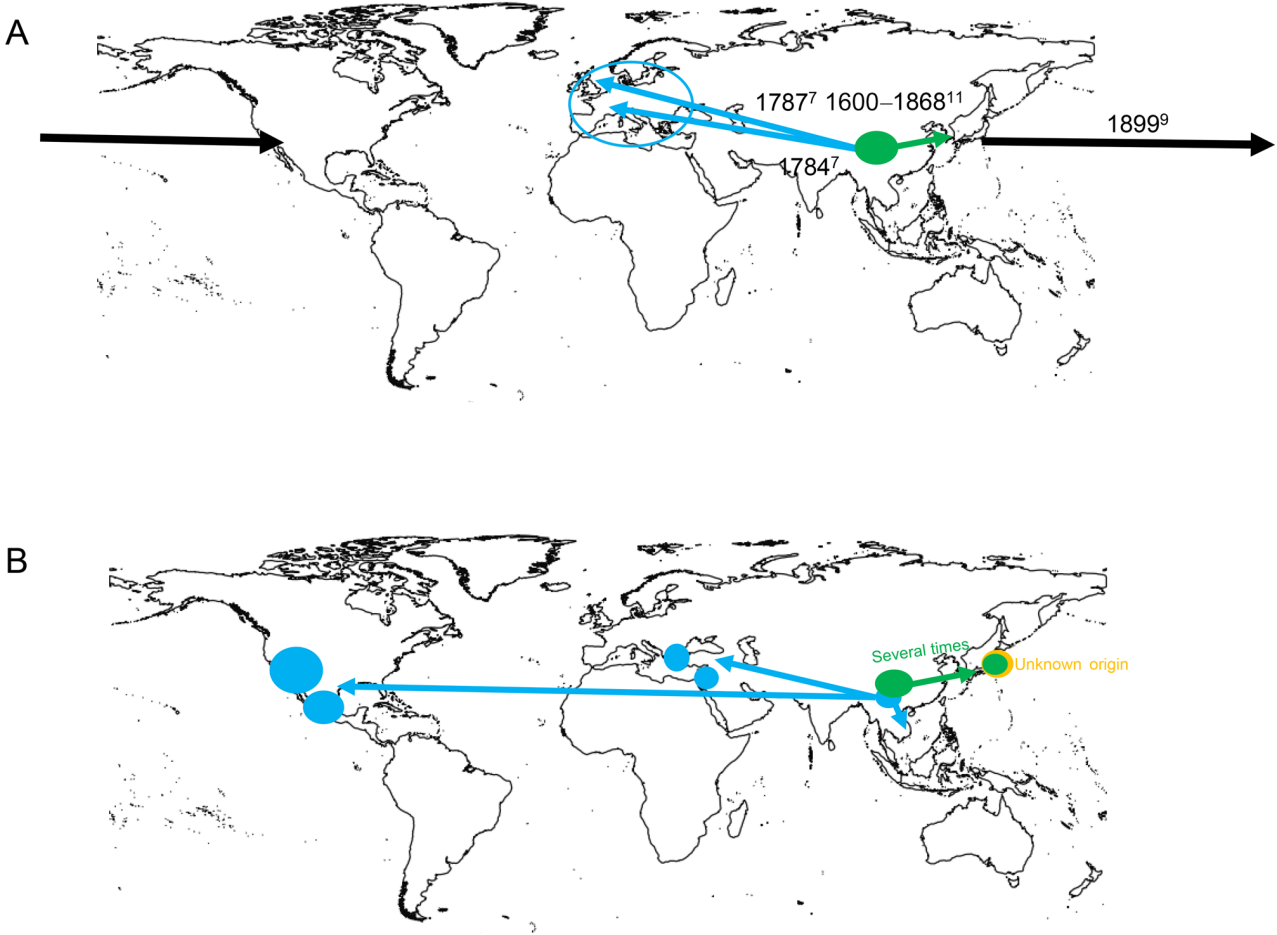
The spread of loquat from its origins in China to other parts of the world (**A**: Propagation by citation) (**B**: Propagation proposed by this study). The base map was produced using SimpleMappr (https://www.simplemappr.net/). Dark green circles indicate Group 1, dark blue circles indicate Group 2, and orange circles indicate Group 3.

Loquat (*Eriobotrya japonica* (Thunb.) Lindl.) is a diploid species (2*n* = 34)^1^ in the family Rosaceae, subfamily Spiraeoideae, tribe Pyreae, subtribe Pyrinae^2,3^. These fruit trees are cultivated in temperate and subtropical zones worldwide. Among the 20 species in the genus *Eriobotrya*, *E. japonica* is the only species used commercially for fruit production^4^. The origin of loquat is reportedly southeastern China^5,6^. In 1784, loquat was transferred from China to the National Garden in Paris, France, and in 1787 from China to the Royal Botanic Gardens at Kew, England^7^. Subsequently, loquat was grown on the Riviera, in Malta, French North Africa, and the Near East, where the fruit began to appear in local markets^8^. There is also a record that it was introduced to California from Japan in 1899 (Fig. 1A), but the details are unknown^9^.

In Japan, loquat seeds have been discovered in excavations of strata from the Yayoi period (300 B.C.–300 A.D.), when rice cultivation technology was introduced from the continent. Loquat was first described in Japan in 762 in the Shōsōin archives, and was also recorded in the Nihon Sandai Jitsuroku (the English translation is “The True History of Three Reigns of Japan”) in 901^10^. Because the current Japanese cultivars were most likely introduced from China about 150–400 years ago (see below), the descendants of the Japanese loquat that predated this introduction are now likely growing as wild strains (wild plants that are not used for commercial cultivation are defined as “strains”). Two possibilities (not necessarily mutually exclusive) have been proposed for the origin of these wild strains^11^: they may have been introduced from China in ancient times by human activity, or they may have been originally indigenous to Japan. The first possibility is supported by the fact that loquat has no native Japanese name, but only a Sino-Japanese name (a Japanese word of Chinese origin)^10^. On the other hand, because some wild strains have been found mixed with native plants throughout Japan and are scattered without forming colonies, and cultivars have not been documented to become wild, wild strains are likely indigenous in Japan^12^. Loquat that grew wild in Japan may not have been actively cultivated for a long time because of the small size and thin flesh of its fruit^12^, which may explain the lack of a native Japanese name.

Although loquat has existed in Japan for more than 1000 years, the current Japanese cultivars were probably bred and spread from seeds introduced from China in the Edo period (1600–1868)^11^. Later, the number of cultivars increased through crossbreeding and bud sport mutations, mainly from ‘Mogi’, ‘Tanaka’, and ‘Kusunoki’.

There are still many unknowns in the transmission of loquat, and elucidation of genetic relationships within this species is important and interesting in loquat breeding research. Loquat genetic diversity has been analyzed with SSR markers^13,14^ and SNP markers^15^, but the number of markers was small. Genome-wide analysis is crucial for accurate evaluation of genetic diversity. Compared to these conventional methods, whole genome resequencing is the most effective method for studying genetic diversity. However, the cost has not decreased sufficiently to analyze a large number of individuals with whole genome resequencing. Restriction site–associated DNA sequencing (RAD-Seq) is an inexpensive method that can analyze a large number of individuals, and it can analyze a large number of markers compared to conventional methods. We have used RAD-Seq to study the genetic diversity of citrus^16,17^, firefly^18^, Japanese pepper^19^, and razor clam^20^, to construct the linkage map of bronze loquat^21^, and to study the phylogenetic relationships among Aurantioideae (citrus and its relatives)^22^. In this study, we used genome-wide SNP markers obtained by RAD-Seq to analyze the genetic diversity and structure of wild loquat strains from various parts of Japan; Japanese cultivars; and cultivars and strains introduced from China, Vietnam, Israel, Greece, the United States, and Mexico.

## Results

### Variant detection by mapping of RAD‑Seq data

The double-digest RAD-Seq (ddRAD-Seq) of the 95 samples (Table 1) generated over 2.8 gigabases with 54.6 million single-ended 51-bp reads. Quality-based filtering yielded 95 samples with an average of 0.57 million reads (maximum, 1.1 million reads; minimum, 0.22 million reads) (Supplementary Table S1). The Stacks program constructed new loci with an average coverage depth of 14 times (Supplementary Table S2). The following analysis was performed using data from 1,822 variant sites.

**Table 1:** Loquat accessions used in this study.

Sample No	Accession name	Origin^x^		Year of introduction	JP number	Group
1	Wanhong	China	Cultivar	1986	203047	1
2	Hongganben	China	Cultivar	1984	202861	1
3	Dayeyangdun	China	Cultivar	1987	203034	1
4	Dahongpao	China	Cultivar	1987	202855	1
5	Changhong-3	China	Cultivar	1984	203042	1
6	Houshanwanshou	China	Cultivar	1989	202862	1
7	Baisha	China	Cultivar	1984	118665	1
8	Xiyeyangdun	China	Cultivar	1987	203038	1
9	Baozhu	China	Cultivar	1987	203033	1
10	Huabao-2	China	Cultivar	1983	202863	1
11	Guangdong^y^	China	Cultivar	1973	175204	1
12	Xialoubaimi	China	Cultivar	1989	203049	1
13	Xialou	China	Cultivar	1989	203048	1
14	Jiajiao	China	Cultivar	1983	202866	1
15	Dazhong	China	Cultivar	1989	175200	1
16	Bahong	China	Cultivar	1989	203041	1
17	Qingzhong	China	Cultivar	1983	202885	1
18	Shanghaipipa^y^	China	Cultivar	1989	203045	1
19	Baiyu	China	Cultivar	1983	175202	1
20	Biqibai	China	Cultivar	1983	115579	1
21	Meihuaxia	China	Cultivar	1989	203044	1
22	Amakusagokuwase	Japan	Cultivar	1974	175201	1
23	Amakusawase	Japan	Cultivar	1974	115578	1
24	Amamishiro	Japan	Cultivar	1990	203096	1
25	Fukuharawase	Japan	Cultivar	1975	172437	1
26	Fukujyuin	Japan	Cultivar	1975	172438	1
27	Fusahikari	Probably of 'Mizuho' × x'Tanaka' bred by SPHI, Japan	Cultivar	1982	172439	1
28	Hondawase	Probably of seedling of open-pollinated 'Mogi', Japan	Cultivar	1952	174614	1
29	Ikeda	Japan	Cultivar	1974	176930	1
30	Kusunoki	Japan	Cultivar	1970	113114	1
31	Mizuho	Probably of 'Tanaka' × 'Kusunoki' bred by NIFTS, Japan	Cultivar	1970	175196	1
32^z^	Mogi	Japan	Cultivar	1952	171486	1
33^z^	Mogi	Same as No. 32	Cultivar	1952	171486	1
34^z^	Mogi	Same as No. 32	Cultivar	1952	171486	1
35^z^	Mogi	Same as No. 32	Cultivar	1952	171486	1
36^z^	Mogi	Same as No. 32	Cultivar	1952	171486	1
37	Morimoto	Probable bud sport mutant of 'Tanaka', Japan	Cultivar	1974	175195	1
38	Moriowase	Probable bud sport mutant of 'Mogi', Japan	Cultivar	1963	176755	1
39	Nagasakiwase	Probably of Mogi' × 'Hondawase' bred by AFTDC in 1976, Japan	Cultivar	-	171484	1
40	Nagasakiwase	Same as No. 39	Cultivar	-	171484	1
41	Natsutayori	Probably of 'Nagasakiwase' × 'Fukuharawase' bred by AFTDC in 2009, Japan	Cultivar	-	–	1
42	Obusa	Probably of 'Tanaka' × 'Kusunoki' bred by NIFTS, Japan	Cultivar	1973	113116	1
43^z^	Shiromogi	Probable seedling from an open-pollinated 'Mogi' seed irradiated with gamma rays bred by AFTDC in 1982, Japan	Cultivar	-	118667	1
44^z^	Shiromogi	Same as No. 43	Cultivar	-	118667	1
45	Suzukaze	Probably of 'Kusunoki' × 'Mogi' bred by AFTDC in 1999, Japan	Cultivar	-	227924	1
46	Tanaka	Japan	Cultivar	1954	171485	1
47	Togoshi	Probably of 'Mogi' × 'Tanaka' bred by SPHI, Japan	Cultivar	1970	174612	1
48	Toi	Japan	Cultivar	1973	176931	1
49	Tomifusa	Probably of 'Tsukumo' × 'Mizuho' bred by SPHI in 1989, Japan	Cultivar	-	203052	1
50	Tsukumo	Prpbably of 'Mogi' × 'Tanaka' bred by NIFTS, Japan	Cultivar	1970	175197	1
51	Yougyoku	Probably of 'Mogi' × 'Morimoto' bred by AFTDC in 1999, Japan	Cultivar	-	227925	1
52	Kawatana mamebiwa No. 1	Collected in Nagasaki, Japan	Strain	2004	–	1
53	Mamebiwa	Collected in Nagasaki, Japan	Strain	1973	117499	1
54	Oita 4	Collected in Oita, Japan	Strain	1975	227943	1
55	Sado 2	Collected in Niigata, Japan	Strain	1977	227952	1
56	Sado 3	Collected in Niigata, Japan	Strain	1977	227953	1
57	Tsushima 12	Collected in Nagasaki, Japan	Strain	1977	–	1
58	Tsushima 15	Collected in Nagasaki, Japan	Strain	1977	–	1
59	Yamaguchi 7S-176	Collected in Yamaguchi, Japan	Strain	1978	–	1
60	Advance	USA	Cultivar	1972	169063	1
61	Fukui 1	Collected in Fukui, Japan	Strain	1976	227947	3
62	Fukui 2	Collected in Fukui, Japan	Strain	1976	227948	3
63	Fukui 3	Collected in Fukui, Japan	Strain	1976	227949	3
64	Fukui 4	Collected in Fukui, Japan	Strain	1976	227950	3
65	Fukui 5	Collected in Fukui, Japan	Strain	1976	227951	3
66	Oita 1	Collected in Nagasaki, Japan	Strain	1975	227940	3
67	Oita 2	Collected in Oita, Japan	Strain	1975	227941	3
68	Oita 6	Collected in Oita, Japan	Strain	1975	227945	3
69	Sado 10	Collected in Niigata, Japan	Strain	1977	227959	3
70	Sado 5	Collected in Niigata, Japan	Strain	1977	227955	3
71	Sado 7	Collected in Niigata, Japan	Strain	1977	227956	3
72	Vietnam loquat Col. No. 97–1	Vietnam	Strain	1997	227933	2
73	Vietnam loquat Col. No. 97–3	Vietnam	Strain	1997	227934	2
74	Vietnam loquat Col. No. 97–4	Vietnam	Strain	1997	227935	2
75	Vietnam loquat Col. No. 97–6	Vietnam	Strain	1997	227936	2
76	Vietnam loquat Col. No. 97–7	Vietnam	Strain	1997	227937	2
77	Vietnam loquat Col. No. 97–8	Vietnam	Strain	1997	227938	2
78	Akko1	Israel	Cultivar	1986	203039	2
79	Akko13	Israel	Cultivar	1986	203040	2
80	Big Jim	USA	Cultivar	1990	203053	2
81	Heads Mamuth	Israel	Cultivar	1987	203036	2
82	Zikim	Israel	Cultivar	1986	203050	2
83	Success	Israel	Cultivar	1987	203037	2
84	Yehuda	Israel	Cultivar	1986	203046	2
85	Zrifin8	Israel	Cultivar	1986	203051	2
86	Greece loquat Col. No. 87–70	Greece	Strain	1987	203095	2
87	Greece loquat Col. No. 87–58	Greece	Strain	1987	178592	2
88	Greece loquat Col. No. 87–67	Greece	Strain	1987	203092	2
89	Greece loquat Col. No. 87–68	Greece	Strain	1987	203093	2
90	Greece loquat Col. No. 87–69	Greece	Strain	1987	203094	2
91	Champagne	USA	Cultivar	1972	169064	2
92	Gold Nugett	USA	Cultivar	1972	116933	2
93	Mexican loquat No. 1	Mexico	Strain	1978	176756	2
94	Mexican loquat No. 2	Mexico	Strain	1978	176757	2
95	Mexican loquat No. 3	Mexico	Strain	1978	176758	2

^z^Technical replicates. ^y^Name derived from the place name in China (in Japan, it is used as the name of the accession). ^x^AFTDC:Agricultural and Forestry Technical Development Center, Nagasaki Prefectural Government. SPHI:Southern Prefectural Horticulture Institute, Chiba Prefectural Agriculture and Forestry Research Center. NIFTS: NARO Institute of Fruit Tree Science.

### Genetic structure

**Figure 2:**
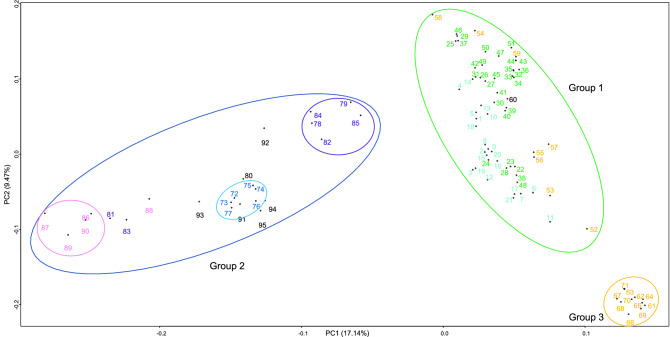
Principal component analysis (PCA) of *Eriobotrya japonica* accessions in the first two components based on 1822 SNP markers. The color of the sample number indicates the country or region where the sample was collected. Cyan represents germplasm resources and cultivars from China, darkgreen represents cultivars from Japan, orange represents germplasm resources from Japan, dark blue represents germplasm resources from Vietnam, purple represents cultivars from Israel, pink represents germplasm resources from Greece, black represents germplasm resources and cultivars from North America. Figure was generated using R software (version 4.1.1).

**Figure 3:**
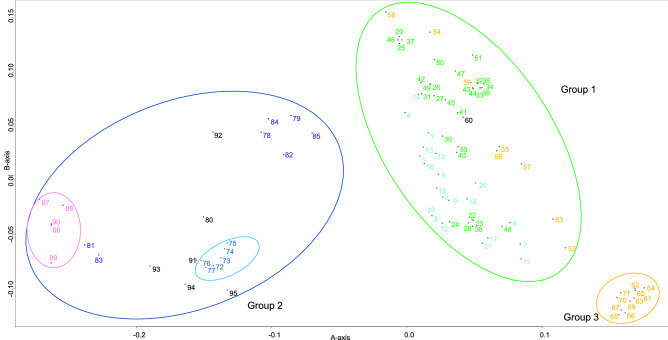
Multidimensional scaling of *Eriobotrya japonica* accessions using 2-dimensional data based on 1822 SNP markers. The color scheme is the same as in Fig. 1. Figure was generated using R software (version 4.1.1).

In principal component analysis (PCA), the contributions of the first and second principal components were 17.1% and 9.47%, respectively (Fig. 2). The first principal component separated the accessions into three major groups: (1) Japanese and Chinese cultivars and some Japanese wild strains, (2) cultivars and strains from Vietnam, Israel, Greece, USA, and Mexico, and (3) other Japanese wild strains. The second principal component separated group 3 from the other groups. Wild strains from different parts of Japan were divided into two groups: some formed a separate group (group 3) and some belonged to group 1. Multidimensional scaling (MDS) analysis also divided the accessions into three major groups (Fig. 3) and supported the grouping by PCA.

PCA and MDS analyses indicated the presence of subgroups. In group 1, most of the Japanese cultivars were in the upper right part, and the Chinese cultivars and some of the Japanese cultivars (22–24, 28, 38, and 48) were in the lower left part. In group 2, with some exceptions, country-specific subgroups could be discerned: Greek subgroup (86, 87, 89, and 90), Israeli subgroup (78, 79, 82, 84, and 85), and Vietnamese subgroup (72–77). Accessions from the USA and Mexico (80, 91 and 93–95) seem to be close to the Vietnamese subgroup. One of the US cultivars (60) belonged to group 1.

**Figure 4:**
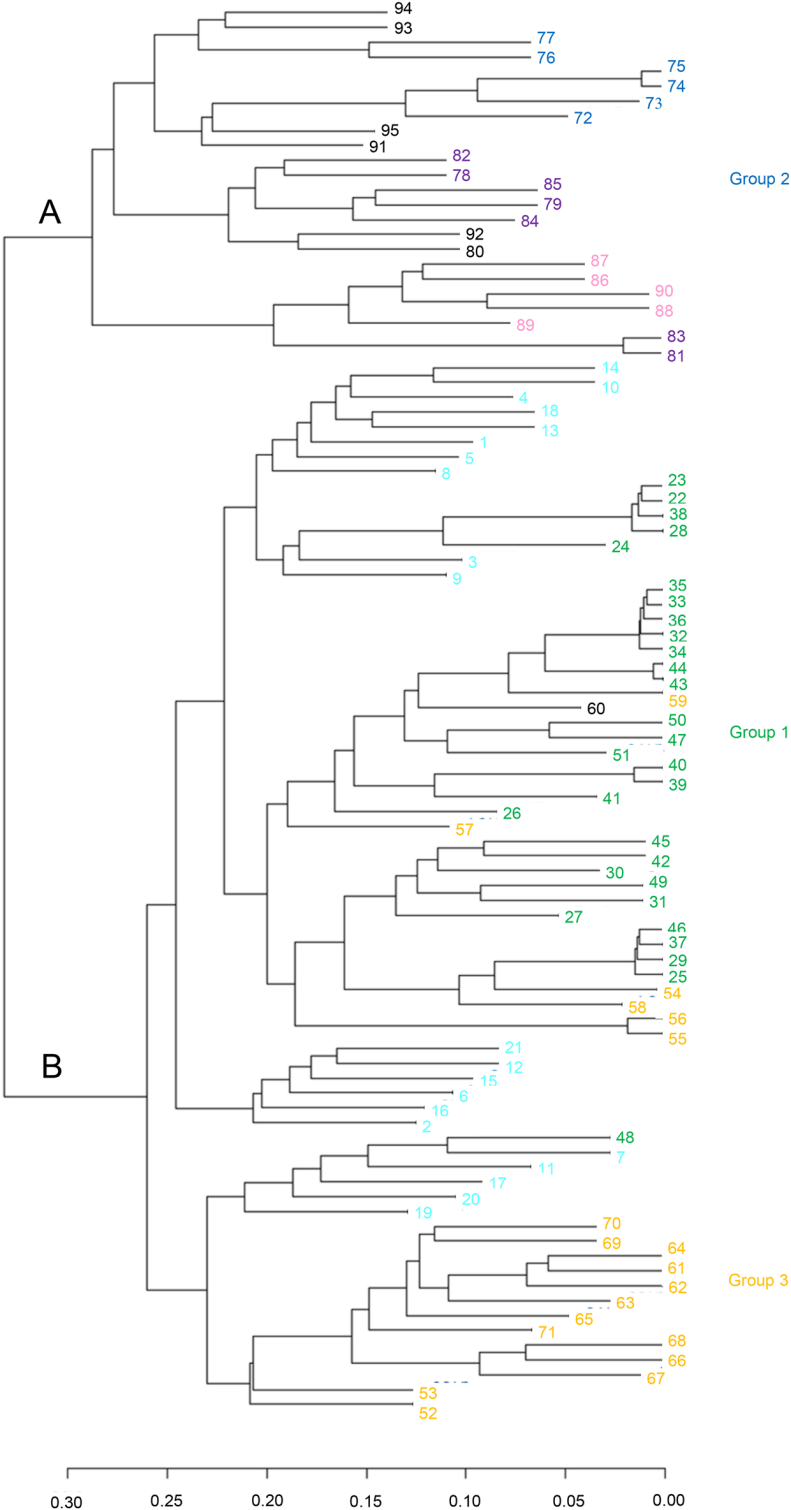
Cluster analysis of *Eriobotrya japonica* accessions. The color scheme is the same as in Fig. 2. Figure was generated using R software (version 4.1.1).

Cluster analysis was performed using a pairwise distance matrix. The cluster analysis identified the two clades (A and B) (Fig. 4). Group 2 was placed in clade A, and group 1 and group 3 were placed in clade B.

**Figure 5:**
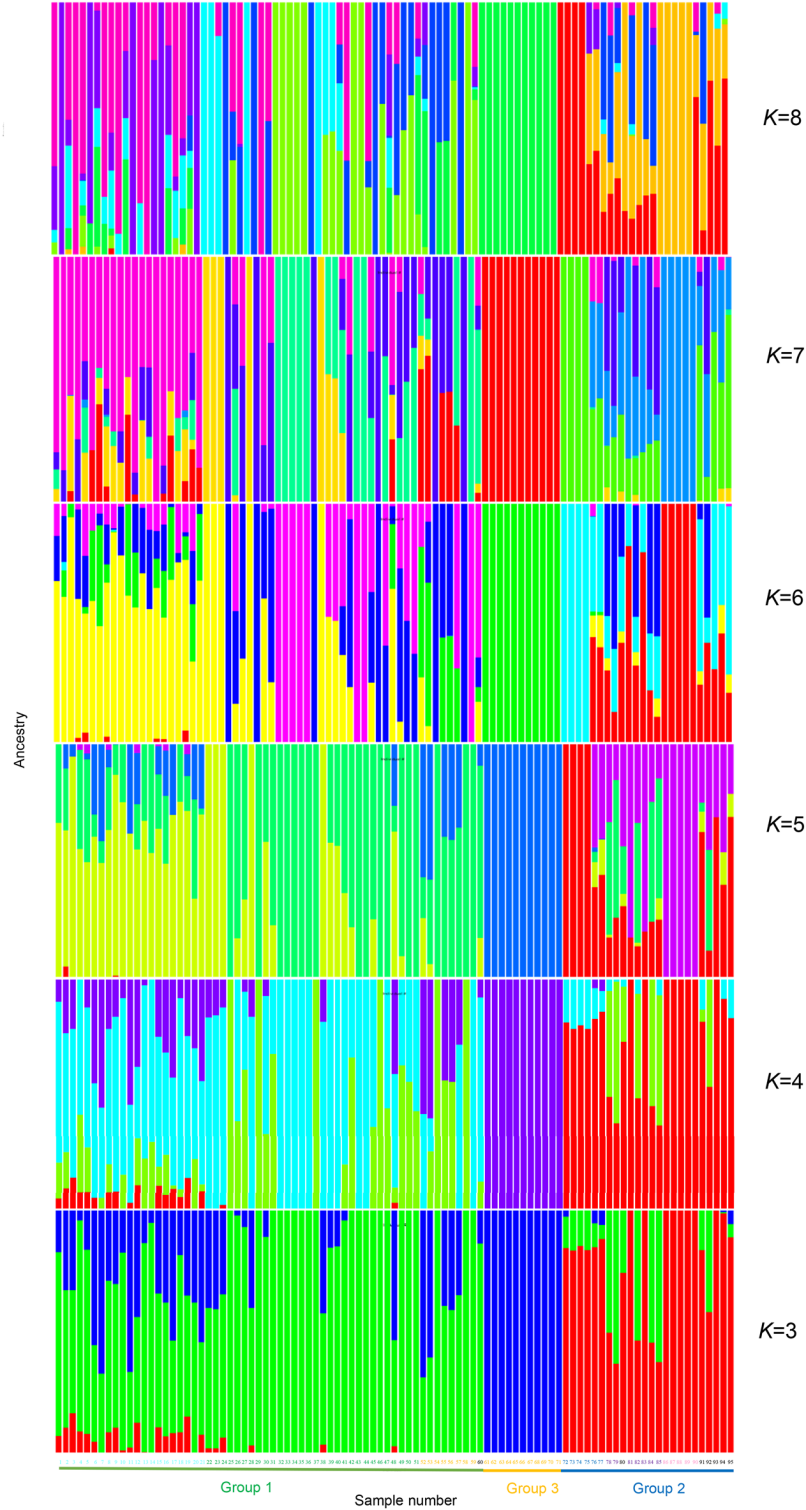
Admixture analysis of *Eriobotrya japonica* accessions. Admixture plots at *K* = 3 to 8 are shown. The colors for the sample numbers are the same as in Fig. 2. The figure was generated using R software (version 4.1.1).

Admixture analysis performed with the number of ancestral populations (*K*) ranging from 1 to 10 (Fig. 5, Supplementary Fig. S1) clearly divided the accessions into three groups, which strongly supports the PCA and MDS results. Reflecting the presence of subgroups as described above, the most likely number of ancestral populations was 7 (Supplementary Fig. S1). No gene flow from other groups was detected in group 3, which consisted of Japanese wild strains.

### Population genetic statistics

Estimation of the degree of genetic diversity among the three groups using the *F*_*st*_ values (Table 2) showed that groups 1 and 3 were the genetically closest pair, as indicated by the lowest *F*_*st*_ value, and groups 2 and 3 were the genetically most distant pair. The mean values of nucleotide diversity (π) and mean expected heterozygosity (*He*) were highest in group 2, followed by group 1 (Table 3, Supplementary Table S3). The mean value of inbreeding coefficient (*F*_*is*_) was lowest in group 3, whereas the *F*_*is*_ values for groups 2 and 3 were similar to each other.

**Table 2:** Pairwise *F*_*st*_ values among the three groups.

	Group 2	Group 3
Group 1	0.103	0.0798
Group 2		0.202

**Table 3:** Population genetic statistics.

	Mean expected heterozygosity	Mean value of π	Mean measure of F_*IS*_
Group 1	0.243	0.245	0.177
Group 2	0.288	0.295	0.178
Group 3	0.158	0.167	0.100

The heterozygosity of each individual was calculated (Supplementary Table S4). Twenty-five individuals exhibited relatively low heterozygosity with a value below 0.15. All 11 individuals (61–71) in group 3 and 6 Japanese wild strains (52–54, and 57–59) in group 2 were in this low heterozygosity group.

### Elucidation of the process of asexual reproduction

Several cultivars have been generated through asexual reproduction, as in the case of a bud sport mutation. To examine whether the parent cultivar and its possible child cultivar were derived from a single tree by a somatic mutation, we checked the conservation of heterozygosity using pairwise alignments (Table 4, Supplementary Fig. S2). The conservation ratio of heterozygous sites between technical replicates was above 90%, which is the criterion^17^ for determining the combinations of the parent–child cultivars derived by asexual reproduction. Consistent with the available records^23^, ‘Tanaka’ (46) was a parent of ‘Morimoto’ (37). Heterozygous sites were conserved among ‘Amakusagokuwase’ (22), ‘Amakusawase’ (23), and ‘Moriowase’ (38). According to oral tradition, ‘Moriowase’ is the oldest, so ‘Amakusagokuwase’ and ‘Amakusawase’ are its descendants. Although the records^10^ claim that ‘Moriowase’ (38) is a bud sport mutant of ‘Mogi’ (32), our analysis rejected this possibility because the conservation ratio of heterozygous sites between these two cultivars was only 19.7%.

**Table 4:** Heterozygosity conservation in pairs of samples.

		Number of variable sites	Number of conserved heterozygous sites	%
'Mogi' (No. 32)	'Mogi' (No. 33)	237	233	98.3
'Mogi' (No. 32)	'Mogi' (No. 34)	229	223	97.4
'Mogi' (No. 32)	'Mogi' (No. 35)	248	239	96.4
'Mogi' (No. 32)	'Mogi' (No. 36)	250	243	97.2
'Tanaka' (No.46)	'Morimoto' (No. 37)	386	375	97.2
'Mogi' (No. 32)	'Moriowase' (No. 38)	594	117	19.7
'Moriowase' (No. 38)	'Amakusagokuwase' (No. 22)	375	356	94.9
'Moriowase' (No. 38)	'Amakusawase' (No. 23)	350	330	94.3
'Amakusagokuwase' (No. 22)	'Amakusawase' (No. 23)	309	302	97.7

## Discussion

Our study analyzed the genetic diversity of 95 cultivars and strains of loquat collected from all over the world. On the basis of the analysis of the population structure of 19 Chinese loquat cultivars and strains by RAD-Seq, Hubei Province in China has been suggested as the center of origin of loquat cultivation^6^. However, the authors have not analyzed the movement of loquats following their introduction from China to other countries and have not verified whether the hypothesis that the cultivation of loquat started in a small area in China^24^ is correct or not. On the basis of the analysis of samples from around the world, we here propose and discuss a more complex history of loquat cultivation (Fig. 1B).

Although genetic diversity analysis using SSR markers revealed no country-specific cultivar clusters^14^, the RAD-Seq analysis used in this study allowed for such clusters to be detected. This was possible because more markers are available in RAD-Seq analysis than in conventional SSR marker analysis. RAD-Seq analysis classified the loquat genetic resources into three groups: (1) Japanese and Chinese cultivars and some Japanese wild strains, (2) Vietnamese, Israeli, Greek, US, and Mexican cultivars and strains, and (3) other Japanese wild strains.

Group 2 had the highest mean values of π and *He* (Table 3). The *F*_*st*_ values (Table 2) showed that group 2 is well separated from groups 1 and 3, which may be due to differences in their place of origin, as discussed below. The *F*_*is*_ value of group 2 was higher than that of the Japanese wild strains (Table 3), which may reflect the fact that group 2 plants have been grown and crossbred by humans.

Group 2 was genetically separated from the Japanese and Chinese loquats. Blasco et al.^25^ reported that SSR and S-allele markers can distinguish European cultivars from other cultivars. Our results are in good agreement with the above study. Morton^8^ reported that loquat genetic resources were introduced to the West from China and Japan, but our study does not agree with this report. When plants are preserved in botanical gardens, records are well kept. However, for commercial use, records are not always kept, because the purpose of plant introduction is different. Group 2 plants may have been introduced to the West for commercial cultivation in a different way than they were introduced to botanical gardens.

The place of origin for group 2 may be in China or Japan, although our analysis failed to find any cultivars or strains in group 2 that originated from China or Japan. Unfortunately, the cultivars and strains from China analyzed in this study are not representative of all loquat cultivars from China, and we were unable to analyze cultivars and strains from southern China, such as Yunnan, Guangdong, and Guangxi. Wang et al.^6^ reported that ‘Younan’ from Guangdong has a close genetic relationship with cultivars from Spain, Italy, and the USA. This suggests that genetic resources from particular regions of China may have been introduced to the West.

The fact that the Vietnamese strains belong to group 2 suggests two possibilities. One is that group 2 originated from Southeast Asian countries other than China, including Vietnam, in particular because cultivars from the USA and strains from Mexico were genetically similar to strains from Vietnam. Interestingly, when group 2 was analyzed in detail, we detected the presence of an Israeli subgroup and a Greek subgroup. They may have originated in Southeast Asian countries other than Vietnam. The second possibility is that cultivars or stains introduced from China and other countries to the USA or France (the former colonial master of Vietnam) were further introduced to Vietnam. Researchers who collected the strains in Vietnam told us that these strains were not used for edible fruit, even though they grew near houses. These plants may have been cultivated in Vietnam as ornamental trees or to be offered to Westerners. Thus, group 2 cultivars are likely to originate from various places.

The differences in taste preferences between the Greek, Israeli, American, and Mexican people and the Japanese and Chinese people may have influenced cultivar differentiation. Indeed, many cultivars and strains from Greece, Israel, USA, and Mexico have higher acid content than those from Japan and China^26,27^. However, differences in taste preferences need to be studied in detail in the future. Although group 2 strains grow in Vietnam, the Vietnamese most likely prefer to eat group 1 fruits because they do not eat group 2 fruits. In Vietnam, loquat imported from East Asia is commercially available.

In group 1, the mean values of π and *He* were lower than in group 2 but higher than in group 3 (Table 3). The *F*_*st*_ values indicated that group 1 is genetically closer to group 3 than to group 2 (Table 2). The *F*_*is*_ value of group 1 was higher than that of the Japanese wild strains (Table 3), which may reflect the fact that group 1 plants, like group 2 plants, have been grown and crossbred by humans.

The PCA and MDS analyses placed Japanese and Chinese cultivars in the center of the group 1 cluster. The present Japanese cultivars originated from excellent Chinese cultivars introduced at the Edo period (1600–1868) and were further improved in Japan^11^. The detection of group 1 reflects this proposed history. The cultivars were believed to have been improved through crossbreeding and bud sport mutations, using mainly ‘Mogi’, ‘Tanaka’, and ‘Kusunoki’. However, some samples (22–24, 28, 38, and 48) were not related to these three cultivars. Other cultivars introduced at the Edo period or earlier or gene flow from Japanese wild strains may have contributed to the formation of these six cultivars.

With the exception of these six samples, the central part of the cluster in group 1 was divided into a subcluster of Japanese cultivars and a subcluster of Chinese cultivars. This separation may reflect differences in the breeding process between China and Japan, where mainly ‘Mogi’, ‘Tanaka’, and ‘Kusunoki’ were used. The slight difference in taste preferences between the Japanese and Chinese may have influenced the formation of the two subclusters, but further research is needed to address this issue.

The PCA and MDS analyses placed the Japanese wild strains at the periphery of group 1. Because group 1 contains Chinese cultivars, the wild strains in this group are closely related to plants introduced to Japan from China. These wild strains may be related to plants introduced before the development of excellent cultivars in China, as well as being related to plants described in ancient Japanese documents. Although other possibilities exist for the five wild strains (52, 53, and 55–57) as discussed below, two strains (54 and 58) are possible descendants of plants introduced to Japan before the Edo period. Analysis of wild strains in China could help to solve this problem, but unfortunately we were unable to analyze them. Among these wild samples, sample 59 was closer to cultivars such as Mogi (32–36, 43, 44, 47), which may be related to cultivar escape.

PCA and MDS analyses placed the Japanese and Chinese cultivars and the Japanese wild strains at the bottom of the cluster in group 1. Admixture analysis suggested that gene flow from isolated Japanese wild strains in group 3 may have occurred in these plants. The finding that gene flow from group 3 may have occurred in Chinese cultivars suggests that plants belonging to group 3 may be present in China. Alternatively, these Chinese cultivars may have been placed here under the influence of populations not analyzed in this study, rather than group 3. Gene flow from group 3 may have occurred to Japanese cultivars (22–24, 28, 38, and 48) and wild strains (52, 53, and 55–57). It is interesting that genetic resources belonging to group 3 are not suitable for edible use and do not have useful traits that can be used to develop new varieties, such as disease resistance.

There are two possibilities for the origin of group 3 plants growing in Japan. The first possibility is that these plants are indigenous to Japan that have not been introduced by human activities. Genetic diversity analysis using SSR markers has demonstrated that wild strains and cultivars in Japan are genetically different^14^, and our data support this conclusion. Group 3 plants growing in Japan had the lowest mean values of π and *He* (Table 3). The *F*_*st*_ values indicated that group 3 was genetically closer to group 1 than to group 2 (Table 2). A notable feature of group 3 was that the lowest *F*_*is*_ value (Table 3), suggesting that humans may not have played a role in the formation of this group. Admixture analysis detected no gene flow from other groups to group 3, even though groups 1 and 3 were genetically close, suggesting that the ancestors of group 3 were not introduced by humans from China, but were indigenous to Japan. Common plants often grow in laurel forest ecoregions in East Asia. Loquat is found in these ecoregions, and may have been growing in Japan since prehistoric times. If these plants are indigenous to Japan, the problem to be elucidated in the future is whether plants belonging to this group are present in China. The second possibility is that the group 3 plants growing in Japan were brought to Japan by human activities from China over the last few thousand years. Although modern people do not use group 3 plants for edible purposes, we cannot rule out the possibility that people in the past used them for edible purposes. In order to clarify these issues, it would be desirable to test and analyze individuals from southern China, such as those from Yunnan, Guangdong, and Guangxi provinces, as well as from the northern parts of Southeast Asian countries.

In this study, we also examined the contribution of breeding through asexual reproduction, as in the case of bud sport mutations (Table 4). We confirmed the available records of the asexual emergence of ‘Morimoto’ from ‘Tanaka’. We also found that ‘Moriowase’, ‘Amakusawase’, and ‘Amakusagokuwase’ were asexually propagated from a single tree. On the other hand, we rejected the possibility that ‘Moriowase’ originated asexually from ‘Mogi’. The above cultivars determined to have been born by asexual reproduction were very similar in fruit traits to their parent cultivars. Thus, our DNA-level analysis allowed to clarify the origin of some cultivars.

This study demonstrates that RAD-Seq analysis is applicable to the genome analysis of loquat, which has relatively low genetic diversity^14^. The information obtained here can be used for loquat cultivar identification and DNA profiling, and in genetic research and breeding programs.

## Methods

### Plant materials

The 95 cultivars and strains examined in this study are listed in Table 1. Among them, 24 are Japanese cultivars, including 11 produced by crossbreeding. The Japanese cultivar ‘Morimoto’ is probably bud sport mutant of ‘Tanaka’, and ‘Moriowase’ is probably bud sport mutant of ‘Mogi’. We also examined 21 cultivars from China, 19 Japanese wild strains, 6 strains from Vietnam, 8 cultivars from Israel, 5 strains from Greece, 3 cultivars from the United States of America, and 3 strains from Mexico. All plants were grown at the Nagasaki Prefectural Agricultural and Forestry Technology Development Center, Nagasaki, Japan. These genetic resources were introduced to Japan before 1997. The collectors took the permit, which was required at the time, and obtained the owner's permission. Samples 33–36 were technical replicates of sample 32, and samples 40 and 44 were technical replicates of samples 39 and 43, respectively.

### DNA extraction and double-digest restriction site-associated DNA sequencing

Genomic DNA was extracted by the CTAB method^28^, treated with RNase and then with phenol/chloroform. The concentration of DNA was measured with a Qubit dsDNA BR Assay Kit (Invitrogen, MA, USA) and adjusted to 20 ng/µl. Libraries for ddRAD-Seq were prepared using the method of Sakaguchi et al.^29^, which is a modification of the original ddRAD-Seq method^30^. The libraries were sequenced by Macrogen (Seoul, South Korea) in one lane of a HiSeq 2000 (Illumina, San Diego, CA, USA). The reads corresponding to each sample were provided.

### Analysis of ddRAD-Seq data

Adapter sequences and low-quality bases were trimmed using fastp v 0.23.0 with default parameters^31^. The genome sequence of a Chinese cultivar ‘Seventh star’ was used as the reference^32^. The BWA-MEM algorithm of Burrows-Wheeler Aligner (version 0.7.17 r1188)^33^ was used for mapping to the reference genome sequence. The ref_map.pl script implemented in the Stacks package (version 2.55)^34^ was used for SNP identification and genotyping (with-X “populations: − R 0.7—ordered-export—vcf—plink—phylip—phylip-var—write-single-snp—min-maf 0.05”). For the PCA and MDS analyses, genotyping data were created to specify all the samples by assigning one individual per population. Each Obs_Het value from “poples.sumstats_summary.tsv” was used for the heterozygosity of each individual. This program was also used to create pairwise alignments between two individuals by changing the -R option to one.

For statistical analysis, groups separated by PCA and MDS analyses were considered as separate populations in the population map data required for the Stacks package. The denovo_map.pl script was re-performed after modifying the data for the population map (with − r 0.7-X “populations: -k—ordered-export—vcf—plink—phylip—phylip-var—write-single-snp—min-maf 0.05”).

### Principal component analysis, multidimensional scaling analyses and cluster analysis

The SNPRelate package^35^ in the R software environment (version 4.1.1) and the vcf file generated by the denovo_map.pl script were used for PCA and MDS analyses. The program converted the vcf file into a gds (genomic data structure) file and created a PCA diagram. Then, the contribution of each principal component was calculated. At this step, only bi-allelic loci were used. The program also used the gds file to create an MDS diagram. The resulting images were generated with the basic functions of the R software environment. The SNPRelate program plotted the dendrogram by considering the identity by state (IBS) pairwise distance. Images of the results were generated using the basic functions of R software environment.

### Admixture analysis

The PLINK 2 program (version 1.90p6.21)^36^ was used to create the input file for the Admixture program. The admixture program (version 1.3.0)^37^ was used to create a cross between the history of admixture and a hypothetical run with *K* (number of ancestral populations) = 1 to 10. The cross-validation error plots were drawn using the obtained log data. The basic functions of R software environment (version 4.1.1) and the Q estimate file created by the admixture program were used to draw the admixture plots.

## Supplementary Information

Supplementary Information.

## Data Availability

Sequences are available at the DNA Data Bank of Japan Sequence Read Archive (https://www.ddbj.nig.ac.jp/dra/index-e.html; Accession no. DRA013114).
