# Supplementary material for: Genetic diversity of loquat (Eriobotrya japonica) revealed using RAD-Seq SNP markers
Source: Sci Rep. 2022 Jun 23;12:10200. doi: 10.1038/s41598-022-14358-9 (PMC9226044; doi:10.1038/s41598-022-14358-9)
Supplement: Supplementary file 1 — Supplementary Information. [file 41598_2022_14358_MOESM1_ESM.docx]

Genetic diversity of loquat (*Eriobotrya japonica*) revealed using RAD-Seq SNP markers

Yukio Nagano^1,2^, Hiroaki Tashiro^3^, Sayoko Nishi^3^, Naofumi Hiehata^4^, Atsushi J. Nagano^5,6^, Shinji Fukuda^2,3,7*^

*^1^ Analytical Research Center for Experimental Sciences, Saga University, Saga, Japan*

*^2^ The United Graduate School of Agricultural Sciences, Kagoshima University, Kagoshima, Japan*

*^3^ Department of Biological Resource Science, Faculty of Agriculture, Saga University, Saga, Japan*

*^4^Agriculture and Forestry Technical Development Center, Nagasaki Prefectural Government, Nagasaki, Japan*

*^5^ Faculty of Agriculture, Ryukoku University, Otsu, Japan*

*^6^ Institute for Advanced Biosciences, Keio University, Tsuruoka, Yamagata, Japan*

*^7^ Center for Education and Research in Agricultural Innovation, Saga University, Saga, Japan*

*Correspondence to S. F. ([sfukuda@cc.saga-u.ac.jp](mailto:sfukuda@cc.saga-u.ac.jp))

**Supplementary Table S1.** Number of filtered reads.

| Sample No. | Number of filtered reads |
| --- | --- |
| 1 | 595,932 |
| 2 | 819,905 |
| 3 | 679,757 |
| 4 | 807,592 |
| 5 | 628,594 |
| 6 | 796,066 |
| 7 | 215,773 |
| 8 | 746,496 |
| 9 | 767,046 |
| 10 | 614,927 |
| 11 | 706,478 |
| 12 | 537,798 |
| 13 | 891,163 |
| 14 | 638,481 |
| 15 | 792,869 |
| 16 | 572,935 |
| 17 | 524,812 |
| 18 | 570,076 |
| 19 | 567,054 |
| 20 | 225,835 |
| 21 | 525,512 |
| 22 | 589,829 |
| 23 | 462,397 |
| 24 | 370,560 |
| 25 | 449,602 |
| 26 | 293,914 |
| 27 | 375,884 |
| 28 | 404,314 |
| 29 | 612,102 |
| 30 | 560,421 |
| 31 | 403,015 |
| 32 | 290,886 |
| 33 | 229,640 |
| 34 | 245,257 |
| 35 | 303,938 |
| 36 | 337,861 |
| 37 | 485,536 |
| 38 | 827,147 |
| 39 | 563,912 |
| 40 | 518,733 |
| 41 | 312,373 |
| 42 | 296,651 |
| 43 | 527,992 |
| 44 | 526,969 |
| 45 | 341,792 |
| 46 | 748,668 |
| 47 | 393,451 |
| 48 | 285,507 |
| 49 | 472,999 |
| 50 | 405,607 |
| 51 | 509,982 |
| 52 | 1,074,570 |
| 53 | 424,616 |
| 54 | 759,581 |
| 55 | 541,717 |
| 56 | 670,854 |
| 57 | 630,772 |
| 58 | 992,424 |
| 59 | 801,154 |
| 60 | 652,979 |
| 61 | 665,120 |
| 62 | 531,049 |
| 63 | 377,066 |
| 64 | 689,974 |
| 65 | 672,899 |
| 66 | 728,512 |
| 67 | 692,039 |
| 68 | 588,033 |
| 69 | 795,185 |
| 70 | 772,873 |
| 71 | 577,815 |
| 72 | 816,070 |
| 73 | 740,183 |
| 74 | 405,659 |
| 75 | 486,521 |
| 76 | 382,382 |
| 77 | 755,431 |
| 78 | 744,170 |
| 79 | 632,820 |
| 80 | 656,743 |
| 81 | 658,281 |
| 82 | 597,657 |
| 83 | 597,431 |
| 84 | 913,781 |
| 85 | 683,231 |
| 86 | 531,899 |
| 87 | 809,005 |
| 88 | 338,641 |
| 89 | 663,574 |
| 90 | 427,968 |
| 91 | 485,730 |
| 92 | 878,559 |
| 93 | 497,149 |
| 94 | 269,203 |
| 95 | 480,999 |
| Total | 54,464,359 |
| Average reads | 573,309 |

**Supplementary Table S2.** Depth of coverage for each sample.

| Sample No. | Depth of coverage |
| --- | --- |
| 1 | 14.7 |
| 2 | 17.3 |
| 3 | 15.5 |
| 4 | 18.0 |
| 5 | 14.3 |
| 6 | 16.0 |
| 7 | 8.82 |
| 8 | 16.7 |
| 9 | 15.9 |
| 10 | 14.8 |
| 11 | 15.4 |
| 12 | 12.8 |
| 13 | 18.9 |
| 14 | 14.8 |
| 15 | 17.3 |
| 16 | 14.3 |
| 17 | 13.4 |
| 18 | 15.7 |
| 19 | 14.4 |
| 20 | 9.55 |
| 21 | 13.8 |
| 22 | 14.0 |
| 23 | 12.1 |
| 24 | 10.7 |
| 25 | 11.5 |
| 26 | 9.34 |
| 27 | 10.3 |
| 28 | 11.2 |
| 29 | 14.3 |
| 30 | 15.3 |
| 31 | 10.6 |
| 32 | 8.79 |
| 33 | 7.91 |
| 34 | 8.24 |
| 35 | 9.04 |
| 36 | 9.42 |
| 37 | 12.4 |
| 38 | 17.8 |
| 39 | 12.5 |
| 40 | 13.2 |
| 41 | 9.62 |
| 42 | 9.39 |
| 43 | 13.7 |
| 44 | 13.2 |
| 45 | 9.69 |
| 46 | 15.9 |
| 47 | 10.9 |
| 48 | 8.82 |
| 49 | 11.8 |
| 50 | 11.3 |
| 51 | 13.8 |
| 52 | 22.8 |
| 53 | 11.5 |
| 54 | 16.0 |
| 55 | 12.2 |
| 56 | 14.6 |
| 57 | 14.5 |
| 58 | 20.5 |
| 59 | 17.4 |
| 60 | 15.8 |
| 61 | 15.7 |
| 62 | 13.5 |
| 63 | 10.5 |
| 64 | 17.2 |
| 65 | 15.2 |
| 66 | 16.8 |
| 67 | 18.5 |
| 68 | 15.4 |
| 69 | 17.2 |
| 70 | 18.0 |
| 71 | 13.5 |
| 72 | 18.8 |
| 73 | 16.8 |
| 74 | 10.9 |
| 75 | 12.5 |
| 76 | 10.3 |
| 77 | 15.9 |
| 78 | 16.8 |
| 79 | 16.3 |
| 80 | 15.3 |
| 81 | 15.3 |
| 82 | 13.6 |
| 83 | 14.0 |
| 84 | 18.9 |
| 85 | 15.7 |
| 86 | 16.2 |
| 87 | 17.4 |
| 88 | 12.7 |
| 89 | 16.0 |
| 90 | 12.0 |
| 91 | 11.5 |
| 92 | 18.8 |
| 93 | 12.9 |
| 94 | 10.4 |
| 95 | 12.6 |
| Average reads | 14.0 |

**Supplementary Table S3.** Population genetic statistics (Content of the "populations.sumstats_summary.tsv" that was created by Stacks program).

| # Variant positions |  |  |  |
| --- | --- | --- | --- |
| # Pop ID | Group 1 | Group 2 | Group 3 |
| Private | 161 | 296 | 21 |
| Num_Indv | 54.40622 | 21.67174 | 10.2187 |
| Var | 34.92984 | 5.89762 | 1.15433 |
| StdErr | 0.13923 | 0.05661 | 0.02449 |
| P | 0.8269 | 0.78519 | 0.87964 |
| Var | 0.02178 | 0.0247 | 0.02683 |
| StdErr | 0.00348 | 0.00366 | 0.00373 |
| Obs_Het | 0.1996 | 0.22746 | 0.12997 |
| Var | 0.0314 | 0.03625 | 0.04439 |
| StdErr | 0.00417 | 0.00444 | 0.0048 |
| Obs_Hom | 0.8004 | 0.77254 | 0.87003 |
| Var | 0.0314 | 0.03625 | 0.04439 |
| StdErr | 0.00417 | 0.00444 | 0.0048 |
| Exp_Het | 0.24273 | 0.28796 | 0.15811 |
| Var | 0.02845 | 0.03114 | 0.03842 |
| StdErr | 0.00397 | 0.00411 | 0.00447 |
| Exp_Hom | 0.75727 | 0.71204 | 0.84189 |
| Var | 0.02845 | 0.03114 | 0.03842 |
| StdErr | 0.00397 | 0.00411 | 0.00447 |
| Pi | 0.245 | 0.29482 | 0.16656 |
| Var | 0.02897 | 0.03262 | 0.04264 |
| StdErr | 0.00401 | 0.00421 | 0.00471 |
| Fis | 0.17735 | 0.17802 | 0.1001 |
| Var | 0.09907 | 0.1252 | 0.1088 |
| StdErr | 0.13923 | 0.05661 | 0.02449 |
|  |  |  |  |
| # All positions (variant and fixed) | |  |  |
| # Pop ID | Group 1 | Group 2 | Group 3 |
| Private | 161 | 296 | 21 |
| Sites | 700615 | 752920 | 697456 |
| Variant_Sites | 1802 | 1840 | 1925 |
| Polymorphic_Sites | 1579 | 1586 | 890 |
| %Polymorphic_Loci | 0.22537 | 0.21065 | 0.12761 |
| Num_Indv | 53.3277 | 21.64499 | 9.94435 |
| Var | 35.65451 | 5.628 | 1.29821 |
| StdErr | 0.00713 | 0.00273 | 0.00136 |
| P | 0.99955 | 0.99948 | 0.99967 |
| Var | 0.00013 | 0.00017 | 0.00011 |
| StdErr | 0.00001 | 0.00002 | 0.00001 |
| Obs_Het | 0.00051 | 0.00056 | 0.00036 |
| Var | 0.00018 | 0.00021 | 0.00017 |
| StdErr | 0.00002 | 0.00002 | 0.00002 |
| Obs_Hom | 0.99949 | 0.99944 | 0.99964 |
| Var | 0.00018 | 0.00021 | 0.00017 |
| StdErr | 0.00002 | 0.00002 | 0.00002 |
| Exp_Het | 0.00062 | 0.0007 | 0.00044 |
| Var | 0.00022 | 0.00028 | 0.00017 |
| StdErr | 0.00002 | 0.00002 | 0.00002 |
| Exp_Hom | 0.99938 | 0.9993 | 0.99956 |
| Var | 0.00022 | 0.00028 | 0.00017 |
| StdErr | 0.00002 | 0.00002 | 0.00002 |
| Pi | 0.00063 | 0.00072 | 0.00046 |
| Var | 0.00023 | 0.00029 | 0.00019 |
| StdErr | 0.00002 | 0.00002 | 0.00002 |
| Fis | 0.00046 | 0.00044 | 0.00028 |
| Var | 0.00034 | 0.00038 | 0.00033 |
| StdErr | 0.00713 | 0.00273 | 0.00136 |

**Supplementary Table S4.** Heterozygosity of variant positions of each individual.

| Sample No. | Heterozygosity |
| --- | --- |
| 1 | 0.223 |
| 2 | 0.17707 |
| 3 | 0.24367 |
| 4 | 0.23318 |
| 5 | 0.21223 |
| 6 | 0.19808 |
| 7 | 0.19736 |
| 8 | 0.19159 |
| 9 | 0.21614 |
| 10 | 0.21899 |
| 11 | 0.21375 |
| 12 | 0.14303 |
| 13 | 0.18941 |
| 14 | 0.223 |
| 15 | 0.24397 |
| 16 | 0.1089 |
| 17 | 0.24272 |
| 18 | 0.20626 |
| 19 | 0.23146 |
| 20 | 0.19415 |
| 21 | 0.17857 |
| 22 | 0.23495 |
| 23 | 0.22702 |
| 24 | 0.1672 |
| 25 | 0.25568 |
| 26 | 0.15813 |
| 27 | 0.24757 |
| 28 | 0.22878 |
| 29 | 0.25878 |
| 30 | 0.20622 |
| 31 | 0.24605 |
| 32 | 0.21039 |
| 33 | 0.20428 |
| 34 | 0.20702 |
| 35 | 0.20664 |
| 36 | 0.20831 |
| 37 | 0.25754 |
| 38 | 0.23799 |
| 39 | 0.23965 |
| 40 | 0.23757 |
| 41 | 0.20476 |
| 42 | 0.17469 |
| 43 | 0.10911 |
| 44 | 0.11398 |
| 45 | 0.18951 |
| 46 | 0.26397 |
| 47 | 0.13791 |
| 48 | 0.21871 |
| 49 | 0.18962 |
| 50 | 0.22655 |
| 51 | 0.1964 |
| 52 | 0.08726 |
| 53 | 0.04804 |
| 54 | 0.1115 |
| 55 | 0.31946 |
| 56 | 0.31329 |
| 57 | 0.09666 |
| 58 | 0.08237 |
| 59 | 0.11841 |
| 60 | 0.24729 |
| 61 | 0.10542 |
| 62 | 0.11998 |
| 63 | 0.10392 |
| 64 | 0.10887 |
| 65 | 0.13977 |
| 66 | 0.09512 |
| 67 | 0.13011 |
| 68 | 0.10315 |
| 69 | 0.11614 |
| 70 | 0.12353 |
| 71 | 0.09809 |
| 72 | 0.18554 |
| 73 | 0.17131 |
| 74 | 0.15753 |
| 75 | 0.16377 |
| 76 | 0.21646 |
| 77 | 0.19053 |
| 78 | 0.33912 |
| 79 | 0.2839 |
| 80 | 0.28848 |
| 81 | 0.27435 |
| 82 | 0.32788 |
| 83 | 0.2858 |
| 84 | 0.33827 |
| 85 | 0.34426 |
| 86 | 0.14001 |
| 87 | 0.15416 |
| 88 | 0.16944 |
| 89 | 0.2017 |
| 90 | 0.15725 |
| 91 | 0.24813 |
| 92 | 0.32429 |
| 93 | 0.11615 |
| 94 | 0.19625 |
| 95 | 0.14358 |

**Supplementary Figure 1.** Cross-varidation (cv) error plotted against the number of ancestors (*K*) at *K*=1-10.


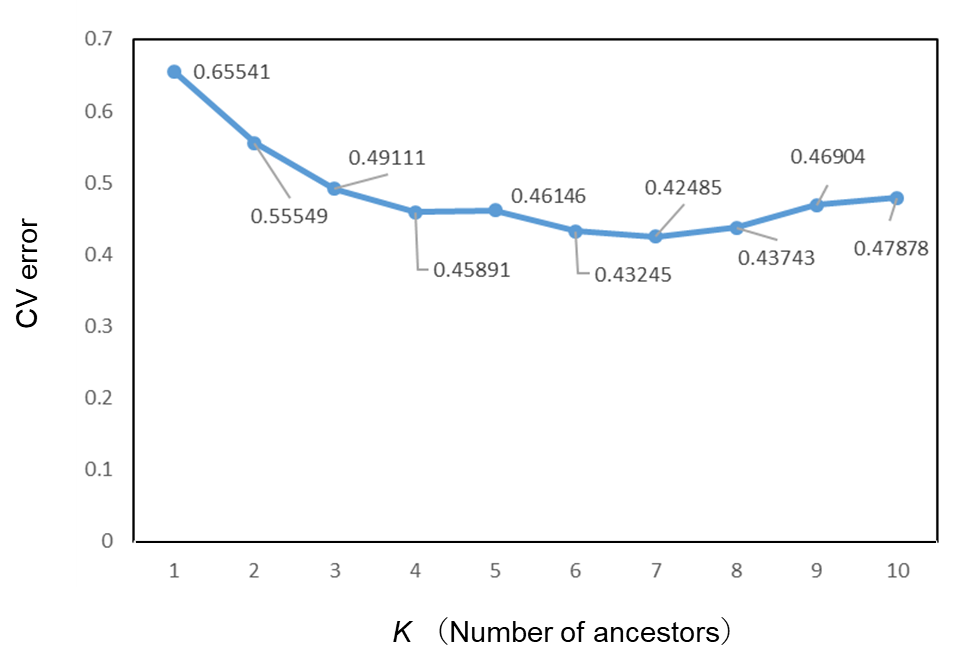


**Supplementary Figure 2.** Pairwise alignments between the variable sequences of 2 samples.

CLUSTAL 2.1 multiple sequence alignment

32 RKWWMKYKWWYKSRRRRSYWYRRMKMYYSRRRGYRSWSRKRWMRWRYYYRKYYRRKRRKM

33 RKWWMKCKWWYKSRRRRSYWYRRMKMYYSRRRRYRSWSRKRWMRWRYYYRKYYRRKRRKM

****** ************************* ***************************

32 YWRYMRWRMRMYYRKYKMYSYKRRMRYRYYRRYYWRYKRYYRYRRYYYYYRRMRKRRRMR

33 YWRYMRWRMRMYYRKYKMYSYKRRMRYRYYRRYYWRYKRYYRYRRYYYYYRRMRKRRRMR

************************************************************

32 RKRKRWSYWYYRRRRYMRYWYMRMYYRYYYKMWRRRWMYYYYYRYMSMYYYRRYRWWSRY

33 RKRKRWSYWYYRRRRYMRYWYMRMYYRYYYKMWGRRWMYYYYYRYMSMYYYRRYRWWSRY

********************************* **************************

32 SYKMYYRYRKWMYYYRMRRRYYRRRYRKKMMYRYYRAYRWYYYRYWYRWWYRRMRYM

33 SYKMYYRYRKWMYYYRMRRRYYRRRYRKKMMYRYYRCYRWYYYRYWYRWWYRRMRYM

************************************.********************

CLUSTAL 2.1 multiple sequence alignment

32 RKWWRMKYKWWYKSRRRSYWYRRMKMYYSRRRMGYRSWRKRWMRWRYYYRYYYYRKRRKM

34 RKWWRMKCKWWYKSRRRSYWYRRMKMYYSRRRMRYRSARKRWMRWRYYYRYYYYRKRRKM

******* ************************* *** **********************

32 YWRRYMRWRMRMYYRKYMYSYKRRMRYRYYRRYYWYKRYYSRRRYYYYRRMWKRRRMRRK

34 YWRRYMRWRMRMYYRKYMYSYKRRMRYRYYRRYCWYKRYYSRRRYYYYRRMWKRRRMRRK

********************************* **************************

32 RKRWSWYYRRRRYRYYMMYYRYYYKMWWRYRRWRMRYYYYYRYMSMYYYRRYRWWSRWYS

34 RKRWSWYYRRRRYRYYMMYYRYYYKMWWAYRRWRMRYYYYYRYMSMYYYRRYRWWSRWYS

**************************** *******************************

32 YKYRYRKWMYYRMRRRYYRRYRKKMMYRKYYRWKYYYYWYRWWYRMRYM

34 YKYRYRKTMYYRMRRRYYRRYRKKMMYRKYYRWKYYYYWYRWWYRMRYM

******* *****************************************

CLUSTAL 2.1 multiple sequence alignment

32 RWWMKKWWYKSRRRRSRYWYRRMKMYYSRRRCGYCCSRWSRKRTWMRWRYYRKYYKYRKR

35 RWWMKKWWYKSRRRRSRYWYRRMKMYYSRRRARYYTSRWSRKRYWMRWRYYRKYYKYRKR

*******************************. * ******* ****************

32 RKMSYWRYYMRMWRMRMYYRKYKMYSYKRRMRYRYRRRYYWRYKRYYRRRRYYYYYRRMW

35 RKMSYWRYYMRMWRMRMYYRKYKMYSYKRRMRYRYRRRYYWRYKRYYRRRRYYYYYRRMW

************************************************************

32 KRRRMRRKRKRWSYWYYRRRRYRYYMRMYYYYYYKMWWRYRRGWRMRYYYYYRYMSMYYY

35 KRRRMRRKRKRWSYWYYRRRRYRYYMRMYYYYYYKMWWRYRRSWRMRYYYYYRYMSMYYY

******************************************.*****************

32 RRYRWWWSRWYSCYAKMYYRYRWMYYYRMRRRYYRRRYRKKMMYRYKYYRWYYYRYWYRW

35 RRYRWWWSRWYSYYRKMYYRYRWMYYYRMRRRYYRRRYRKKMMYRYKYYRWYYYRYWYRW

************ * *********************************************

32 WYRKMYYM

35 WYRKMYCM

****** *

CLUSTAL 2.1 multiple sequence alignment

32 RKWWMKKWRWYKSRRRSYYWYRRMKMYYSRRRMGYCSRWSWRKRWMRWRYYYRYKYYYRR

36 RKWWMKKWRWYKSRRRSYYWYRRMKMYYSRRRMRYYSRWSWRKRWMRWRYYYRYKYYYRR

********************************* * ************************

32 KRRKMYWRYYMRMWRMRMYYRKYMSYKRRMRYRYRRRRYYWRYKRYYRRRRWYYYYYRRM

36 KRRKMYWRYYMRMWRMRMYYRKYMSYKRRMRYRYRRRRYYWRYKRYYRRRRWYYYYYRRM

************************************************************

32 RWKRRMRYRGKRKRWSYWYYRRRKRYRYWYMMYYYYYYYKMWWRYRRGWRMYYYYYRYMS

36 RWKRRMRTRRKRKRWSYWYYRRRKRYRYWYMMYYYYYCYKMWWRYRRSWRMYYYYYRYMS

******* * *************************** *********.************

32 MYYYRRYRWWSRWYSYKYYRYRTWMKYYYRMRRRYRRRYRKYKMMYRYKYYRWYYYRYWY

36 MYYYRRYRWWSRWYSYKYYRYRGWMKYYYRMRRRYRRRYRKYKMMYRYKYYRWYYYRYWY

********************** *************************************

32 RWWWYRYMYM

36 RWWWYRYMYM

**********

CLUSTAL 2.1 multiple sequence alignment

46 RKRKYRSWRRYRRWRYYRYKSRRYRRRRYRYKRMYRYYWYYYKMRYYYSRKKKRSKSRKR

37 RKRKYRSWRRYRRWRYYRYKSRRYRRRRYRYKRMYRYYWYYYKMRYYYSRKKKRSKSRKR

************************************************************

46 YSYMRRWSKSRYRWKRGRKSYYWYYKSRKKRYRMSYYYKYWKWSKCWYYYYSSRRRYYKY

37 YSYMRRWSKSRYRWKRCRKSYYWYTKSRKKRYRMSYYYKYWKWSKTWYYYYSSRRRYYKY

**************** ******* ******************** **************

46 RRYRYMYRYSWYYYMRRYRYRYYSKRRMYRMRYMKMRSRRRYWRYRYYYKYYKRWRRRKS

37 RRYRYMYRYSWYYYMRRYRYRYYSKRRMYRMRYMKMRSRRRYWRYRYYYKYYKRWRRRKS

************************************************************

46 RMRYCYYRRRRYWYYYYMSRRYRYWRSKYYYRRRCMRYYYYYRYWKKRYCWRMSRWYYYK

37 RMRYYYYRRRRCWYYYYMSRRYRYWRSKYYYRRRGMRYYYYYRYWKKRYYWRMSRAYYYK

**** ****** ********************** ************** ***** ****

46 YWSRRYMMRWMRRRYWGYKMKRMYKRYYYYYTMWWRRSWWMRYYYYRRYRRYYRYWYYRR

37 YWSRRYMMRWMRRRYWAYKMKRMYKRYYYYYKMWWRRSWWMRYYYYRRYRRYYRYWYYRR

****************.**************.****************************

46 YKRKSYYRSYWRSRRYRKYYKRMRRYYYRRARRRRYYMRRMYYRWSWYSYYRRRRYWWYY

37 YKRKSYYRSYWRSRRYRKYYKRMRRYYYRRWRRRRYYMRRMYYRWSWYSYYRRRRYWWYY

****************************** *****************************

46 WYKKMYKSRYWRWYYYYMMRRYRYYM

37 WYKKMYKSRYWRWYYYYMMRRYRYYM

**************************

CLUSTAL 2.1 multiple sequence alignment

32 GRCAKATTCTWCAWMKYRKCRCWYGKGSRRGTRRAAASGYYCWAGYRTRMYKCMTYACSA

38 RRTWGMACGYWYWWMKCRKYRYWTRTKCGGRCGARRWSRYYYTTATGCAACGACCTGTGG

* : * *** ** * * . . * ** :. . . ..

32 CRRRATGTTCACCACGAMYTATCACCSRACGWCSWSRAGGKSTCTRGGGWMRCGCRCCCA

38 TRRRRCCYCYRMYRMRRACKRYYCYYGAGYRAYGWCRRARGSYSAARRKTMRYSYRSYYG

*** . . . . .*.* . * .: ** . *. .

32 YYRYAKYTGYGYRRTGRKRRGTTKCMGGYTSGTYGTGWAATRRRTGCCYTTMRMWGTCGA

38 CCGCTKYYRTKTGGYARTGRKKCGSCRKYCSACYAYRWRRYRRGYRYYYYYCGMWAYMTW

:** .*. * . . * *. *. * ** * **.

32 GRTGTTMRMYYRKTTGYMYSYAKRRMRCYRYRYCCCTCTGCTGAGAGCCCCGTGGAGGAT

38 TGGKKKMGMYYRKYYRYCCGTGGARCAATGTATTTACTCAACTGCGAATGTACTATTATC

..* ***** * . . * . . .. . ... . .: .:

32 GAATAAATCTGCGCRGCGTRYYWRYKRAYTACYTCTTCAGRYATACGRRWGYCYYYYRCG

38 TGRYRMRCSCKYTYAKYSWACYWRYKRMTCRTCWYKCSWRRCRKTYARRTRYTTCTTAAR

. . . ****** . . * .: .** * .

32 TRMCRWTCKGGRRRMCRRAGGKRKRWACCTASTTATYTWYGAACGGAAGGATRRCRRYCM

38 CAATRWYMKRKAARCYGGCAKGGTGARYYWMCAYGYTCTTSGRGRSCWRKRAAATAGTTC

** * * .. . .: . .. .. :

32 RYWYMCRMYRYYRAYYGYYKKGCMWWRRTARCTWCMRYYYRYYYMSMYYYRGAGRRYRWT

38 RYWYMYRMYRYYRWYYRYYKKCYCWWRRYWGGAWSMATTCGCTTCCACTYRAWRRACAAC

***** ******* ** **** **** :*.* . **. *

32 WAWSRCTTKTWYATGTASCYAACKACCMGYTTACRCCTYRAATTYTWMTCACKYYYGCTR

38 ACACATKKKCWYRYRCTSYYGGYKGGGCACYWRMRSYYCARMCGCYWMYYCSKTCCRYYG

. . ..* ** :* *.. *. . *. ** ..*

32 MRCRTRCYCRCKYRRTRYRKCGGYATKMTGMYACCGYGAAATTTRCACYKYGRCYAGRWK

38 MGYAYRYYYAYTTARWRYRKYRRYCCKAKRAYGTTCCAGGGCACRTGTTTCRRGYRSRTG

* * * . * **** *. * . *. .... : * . . * * .*

32 YCTTCGYRWCACTTGTTTACYTAGRRCAWAWWYCGTRTTKGAGCMCTTAGTTRM

38 TMYWYKTRWYWGYWSKYYWSYATRGATGWRWAYTAYRCCGAGAACYGYRACCRM

** .. .*:: .* * * . * .... . **

CLUSTAL 2.1 multiple sequence alignment

38 RRWMYYYYWSYWWMKRKYRYSWRKMRRRWSRGYYYRMRRRYYRMYRMRRRKRYYYRTYRY

22 RRWMYYYYWSYWWMKRKYRYSWRKCARRWSRRYYYRMRRRYYRMYRMRRRKRYYCRCYRY

************************ ***** ********************** * ***

38 WRRRWSYSCRRKYMRMRSYRSYYYKYYRYYKYRRKKSRKYRSSKYYRWRRYRRYRRYYYY

22 WRRRWSYSYRRKYMRMRSYRSYYYKYYRYYKYRRKKSRKYRSSKYYRWRRYRRYRRYCYY

******** ************************************************ **

38 YYAWYMWYKKRKMMMYYRKYYYRYRRWSRYRMRRSKMYKYSCYWRYKYRRMRWYYKRSSW

22 YYRWYMWYKKRKMMMYYRKYCYRYRRWSRYRMRASKMYKYSTYWRYKYRRMAWYYKRSSW

** ***************** ************ ******* ********* ********

38 RRRRKRTYRRRRRYRRARYWYYMKRKRYKKSRYYWMYYSRRSKWRRKRRYWYMYRMYRYY

22 RRRRKRAYRRRRRYRRRRYWYYMKRKRYKKSRYYWMYYSRRSKWRRKRRYWYMYRMYRYY

******:********* *******************************************

38 RRWYRYYYWWRRYWYSWSMRYRYWRRKKMKWYRYRYSYYKYWRMSYYRMYAWMYYSKYRY

22 RRWYRYYYWWRRYWYSWSMRYRYWRRKKMKWYRYRYSYYKYWRMSYYRMYGWMYYSKYRY

**************************************************.*********

38 YMYYYRYYYKYRWRYYRRKYMRRYCKRYKRYRRRYRSRKMYWWYKWYYWYYWSKYYWSYR

22 YMYYYRYYYKYRWRYYRRKYMRRYYKRYKRYRRRYRSRKMYWWYKWYYWYYWSKTYWSYA

************************ ***************************** ****

38 WRWYYYYMRRYYRYM

22 WRWYYYYMRAYYRYM

********* *****

CLUSTAL 2.1 multiple sequence alignment

38 RRWMYYYWYWWRMKRKYRYSWRKMRRRWSRGYYYRRRRYYRMYRMRRRKRYYYYYWRRRR

23 RRWMYYYWYWWRMKRKYRYSWRKMRRRWSRRYYYRRRRYYRMYRMRRRKRYYYYYWRRRR

****************************** *****************************

38 SYSRRKMRRSYRRSYYTKYYRYKYRRKKSRKYSSKYYRWRYRRYRMRYYYYRMWYMWYKK

23 SYSRRKMRRSYRRSYYWKYYRYKYRRKKSRKYSGKYYRWRYRRYRMRYYYYRMWYMWYKK

**************** ****************.**************************

38 KMMMYAYRKYYRYRRWRRYRMRRSKMYRKYSMYWRYKYRMRWYKRSSARRRKRYRCRRYR

23 KMMMYGYRKYYRYRRWRRYRMRGSKMYRKYSMYWRYKYRMAWYKRSSGRRRKGYRYRRYR

*****.**************** ***************** ******.**** ** ****

38 RRYWMKRKRYYKKSYYWMYYSRRSWRRKRRYWYMYRMYYYRWYYRYYKYWWRRYWYSRWS

23 RRYWMKRKRYYKKCYYWMYYSRRSWRAKRRYWYMYRMYYYRWYYRYYKYWWRRYWYSRWS

*************.************ *********************************

38 MYRYRYWRRKKMKWYRYCSYYKKYWRMSYYRMYWMYYSKRYYMYYYRYYYKRWYRKYMRR

23 MYRYRCWRRKKMKWYRYGSYYKKYWRMSYYRMYWMYYSKRYYMYYYRYYYKRWYRKYMRR

***** *********** ******************************************

38 YKRYRKRYRRRYRSRMYWYKWYYWGYWSKYWRSYYWKRWYYYYYYRRRRM

23 YKRYRKRYRRGYRSRMYWYKWYYWCYWSKYWRSYTWTRWYYYTYYRRAGM

********** ************* ********* *.***** **** *

CLUSTAL 2.1 multiple sequence alignment

22 RRWMRYWYWWRMKRKYRMYWRKRRWSRRYYYRRRRYYRMYRMRRRKRYYRYRYRRRYSRR

23 RRWMRYWYWWRMKRKYRMYWRKRRWSRRYYYRRRRYYRMYRMRRRKRYYRYRYRRRYSRR

************************************************************

22 KMRRSYRSYYYWKYYRKYYRKKSKYSKYYRWRYRRYRYCYYYRRWYMWYKKKMMYYRKYY

23 KMRRSYRSYYYWKYYRKYYRKKSKYSKYYRWRYRRYRYYYYYRAWYMWYKKKMMYYRKYY

************************************** **** ****************

22 RYKRRRYRMRRASKMYAKYSYWKRYKRMWYKRSSRRRKRRRRYRRRYWYRMKRKRYKSYW

23 RYKRRRYRMRRGSKMYGKYSYWKRYKRMWYKRSSRRRKRRRRYRRRYWYRMKRKRYKCYW

***********.****.****************************************.**

22 MRYYSRSWRKRRYWYMYRMYYYRYYWRYYGYWWRRWSWSMRYRWRRKKKWYYSYKYWRMS

23 MRYYSRSWRKRRYWYMYRMYYYRYYWRYYKYWWRRWSWSMRYRWRRKKKWYYSYKYWRMS

***************************** ******************************

22 YYRMYWMYYSKRYYMYYRYYYRWYRKYMRRKKRYRRRYRSRMYWYKRWYYWYWSKYWSYW

23 YYRMYWMYYSKRYYMYYRYYYRWYRKYMRRKKRYRRGYRSRMYWYKRWYYWYWSKYWSYW

************************************ ***********************

22 RWYYYYYRM

23 RWYYYYYRM

*********
